# Response to Peptide Receptor Radionuclide Therapy in Pheocromocytomas and Paragangliomas: A Systematic Review and Meta-Analysis

**DOI:** 10.3390/jcm12041494

**Published:** 2023-02-13

**Authors:** Antonella Lucia Marretta, Alessandro Ottaiano, Domenico Iervolino, Alessandra Bracigliano, Ottavia Clemente, Francesca Di Gennaro, Roberto Tafuto, Mariachiara Santorsola, Secondo Lastoria, Salvatore Tafuto

**Affiliations:** 1 Department of Clinical and Surgery Oncology Unit, University of Naples “Federico II”, Via S. Pansini 5, 80131 Naples, Italy; 2 SSD Innovative Therapies for Abdominal Metastases, Department of Abdominal Oncology, Istituto Nazionale Tumori di Napoli, IRCCS “G. Pascale”, Via M. Semmola, 80131 Naples, Italy; 3 Pathology Unit, Istituto Nazionale Tumori di Napoli, IRCCS “G. Pascale”, Via M. Semmola, 80131 Naples, Italy; 4 Nuclear Medicine Unit, Istituto Nazionale Tumori di Napoli, IRCCS “G. Pascale”, Via M. Semmola, 80131 Naples, Italy; 5 Sarcomas and Rare Tumours Unit, Istituto Nazionale Tumori di Napoli, IRCCS “G. Pascale”, Via M. Semmola, 80131 Naples, Italy; 6 Department of Neuroscience and Reproductive and Dental Sciences, Division of Neurosurgery, University of Naples “Federico II”, Via S. Pansini 5, 80131 Naples, Italy

**Keywords:** PRRT, pheocromocytomas, paragangliomas, neuroendocrine tumors, meta-analysis, 177Lu-DOTATATE, 90Y-DOTATOC

## Abstract

Introduction. Peptide receptor radionuclide therapy (PRRT) with 177Lu-DOTATATE and 90Y-DOTATOC showed efficacy in the metastatic setting of pheocromocytomas (PCCs) and paragangliomas (PGLs) where no standard therapies have been established. Background. A search of peer-reviewed and English articles reporting on 177Lu-DOTATATE and 90Y-DOTATOC efficacy was performed through Medline and Scopus. A subsequent meta-analysis was performed to evaluate the pooled effect size on disease control rate (DCR) with PRRT. Secondary endpoints were description of patients’ genetic characteristics, hematologic toxicity, and time-to-outcome. The pooled effect was estimated with both a mixed-effects model and a random-effects model. Results. Twelve studies met the criteria for this meta-analysis: ten with 177Lu- and two with 90Y-PRRTs (213 patients). The largest one included 46 patients. Median ages ranged from 32.5 to 60.4 years. When reported, mutations of SDHB were the most frequent genetic alterations. The pooled DCRs were 0.83 (95% CI: 0.75–0.88) and 0.76 (95% CI: 0.56–0.89) for 177Lu- and 90Y-PRRT, respectively. The pooled DCR for PRRT was 0.81 (95% CI: 0.74–0.87). Conclusions. We report an updated and solid estimate of DCR achieved with 177Lu- and 90Y-PRRT in PCCs and PGLs, showing that these therapies can be considered in the multidisciplinary treatment of PCCs and PGLs as alternatives to I-131 MIBG and chemotherapy.

## 1. Introduction

Pheocromocytomas (PCCs) and paragangliomas (PGLs) are tumors derived from adrenal medulla and extra-adrenal paraganglia, respectively, developing in relationship with parasympathetic and sympathetic components of the autonomic nervous system [1]. Their incidence is about 0.6 cases per 100,000 person-years, and the most common symptoms guiding diagnosis are palpitations, hypertension, profuse sweating, and headaches. They are more common in females than males, and have a higher incidence in people over the age of 40 [2]. Hereditary syndromes linked to PCCs and PGLs are von Hippel-Lindau disease (VHL), neurofibromatosis type 1 (NF-1), and multiple endocrine neoplasia, especially type 2 (MEN 2); however, a non-negligible number of PCCs and PGLs are non-functioning, and most of them are sporadic [3]. These features contribute to diagnosis delay. Geographically, PCCs and PGLs have a higher incidence in North America, Europe, and Australia compared to Asia. The tumors are also more commonly found in individuals of northern European descent. Association of clinical, biochemical, and histological findings is the best way to determine PCC and/or PGL malignant potential [4].

Imaging approaches used to identify PCCs and PGLs are based on CT and MRI with a sensibility of 77–98% and 90–100%, and specificity of 29–92% and 50–100%, respectively [5]. The most important functional imaging is PET/CT 68Ga-DOTATATE (DOTA-Tyr3-octreotate), which shows superiority over I131-MIBG SPECT/CT [6], and has an excellent diagnostic power for extra-adrenal sympathetic PGLs and metastatic and multifocal PGLs and PCCs, according to European Association of Nuclear Medicine and Society of Nuclear Medicine and Molecular Imaging (EANM/SNMMI) guidelines [7]. The current diagnostic role of the 131I-MIBG appears to be limited to abdominal paragangliomas and theranostic purposes [6]. Somatostatin receptor imaging is performed with different DOTA-coupled somatostatin agonists, namely DOTA-Tyr3-octreotate (DOTATATE), DOTA-Tyr3-octreotide (DOTATOC), and DOTA-Nal3-octreotide (DOTANOC) [6,7].

There are no widely accepted, standard sequential treatments for PCCs and PGLs. According to international guidelines [4,5,6,7], somatostatin receptor positivity allows the treatment of metastatic PCCs or PGLs with peptide receptor radionuclide therapy, or PRRT (theranostic) [8]. Briefly, PRRT is a treatment modality that has gained great relevance in the management of various neuroendocrine tumors (NETs). The concept of PRRT is based on the selective targeting of somatostatin receptors (SSTRs) that are highly expressed on the surface of NET cells. PRRT leverages the capability of radiolabeled somatostatin analogues to deliver high doses of ionizing radiation directly to the site of the tumor mass. In this way, PRRT effectively targets and destroys the malignant cells while minimizing exposure to normal surrounding tissues. The mechanism of action of PRRT can be divided into two phases. The first phase involves the localization and internalization of the radiolabeled somatostatin analogue in the NET cells. This is achieved through the high-affinity binding of the analogue to the SSTRs on the cell surface. The internalization of the analogue initiates a cascade of intracellular events that culminate in the formation of endocytic vesicles. In the second phase, the radionuclide is delivered to the target tumor cells, releasing a high dose of ionizing radiation. This radiation causes cellular damage by inducing double-stranded DNA breaks and oxidative stress, leading to the death of the cancer cells. Most importantly, the high specificity of the SSTR-targeting mechanism ensures that normal cells with low SSTR expression are spared, minimizing the risk of side effects.

In conclusion, PRRT may offer a highly targeted and effective treatment option for patients with NETs. By leveraging the SSTR-targeting mechanism, PRRT delivers high doses of ionizing radiation directly to the site of the tumor, reducing the risk of systemic toxicity and maximizing therapeutic efficacy. The mechanism of action of PRRT demonstrates the potential of targeted radionuclide therapy as a safe and effective modality in the management of NETs.

In this context, 177Lu-DOTATATE and 90Y-DOTATOC PRRTs have shown good results, particularly in terms of disease control improvement.

This meta-analysis comprised studies focusing on treatment with 177Lu- and 90Y-PRRTs; our principal aim is to provide the scientific community with an updated and solid estimate of DCR (disease-control rate) achievable with these therapeutic options.

## 2. Materials and Methods

The present systematic review and meta-analysis was performed to investigate the role of 177Lu-DOTATATE and 90Y-DOTATOC in the treatment of metastatic PCCs and PGLs. It was conducted according to the recommendations of the Preferred Reporting Item For Systematic Reviews and Meta-Analysis (PRISMA) 2020 statement [9]. The primary outcome was the disease control rate (DCR: percent of complete responses plus partial responses plus stable disease) of patients treated with 177Lu-DOTATATE and 90Y-DOTATOC. According to our internal policies, the institutional review board approval and PROSPERO (International Prospective Register of Systematic Reviews) registration were not required for the systematic literature review. Trial search strategy is described below.

### 2.1. Search Strategy and Trial Identification Criteria

**Figure 1:**
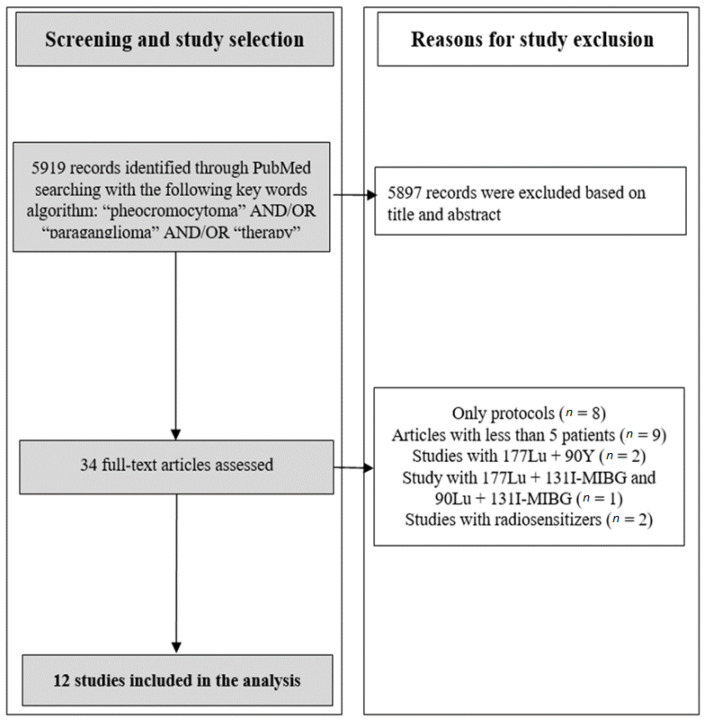
Selection flow-chart of studies included in the meta-analysis.

A search was performed on September 2022 through Medline (PubMed: www.ncbi.nlm.nih.gov/PubMed, accessed on 12 September 2022) and Scopus. Keywords used for searching were “pheochromocytoma” AND/OR “paraganglioma” AND/OR “therapy”. A large time range (2006–2022) was applied. In addition to computer browsing, manuscripts were also checked in the reference section to select further reports. A flow-chart reporting the criteria for study selection and exclusion is depicted in Figure 1. Only peer-reviewed and English papers on 177Lu-DOTATATE and 90Y-DOTATOC efficacy were considered. Manuscripts reporting on fewer than 5 patients were excluded. To avoid spurious and heterogeneous results, studies reporting on multiple PRRT agents and/or plus concomitant chemotherapy or radiosensitizers were excluded.

### 2.2. Data Extraction

The following data were extracted for each study: first author; year of publication; type of study; clinic-pathological and genetic characteristics of patients; type of PRRT agent; treatment characteristics; number of patients enrolled; no. of PRRT cycles; response criteria; best response; hematological toxicities; previous treatments. All data were reviewed and separately computed by three investigators (ALM, DI and AO). Criticisms and discordances were discussed with all authors.

### 2.3. Studies Quality Rating

The quality rating of the methodologies and results of the selected studies was managed by three authors (A.L.M., M.S., and O.C.) to provide a formal quality evaluation as well as a risk-of-bias assessment. To this end, MINORS (Methodological Index for Non-Randomized Studies) criteria [10] and Newcastle–Ottawa Scale [11] were used. Final scores were independently rated by A.O. and D.I., who were blinded to the previous results. Discordances were resolved in consensus discussion involving A.L.M., M.S., O.C., A.O., and D.I. The impact factors (Ifs) of the scientific journals reporting the analyzed articles are publicly available at https://jcr.clarivate.com/jcr/home (accessed on 28 December 2022). They were taken into account after a final evaluation of the studies’ quality. However, as our policy, possible associations between Ifs and quality scores were neither explored nor shown.

### 2.4. Statistical Methods

A meta-analysis was performed in order to evaluate the pooled effect size in response to PRRT agents in pheocromocytomas and paragangliomas. The primary endpoint of the meta-analysis was the disease-control rate (DCR). The description of clinic-pathological and genetic characteristics was a secondary endpoint.

The pooled effect of the various studies was estimated with both fixed- and a random-effects models. The fixed-effects model assumes that all studies being analyzed have the same underlying effect size. This means that any differences in results between studies are solely due to random error or chance, rather than differences in the underlying population or treatment effect. The fixed-effects model uses a weighted average of the study-specific effect sizes to estimate the overall effect size. The random-effects model assumes that the true effect size varies between studies. This may be due to differences in the population, treatment, or study design. The random-effects model uses a more complex model to account for this variability, by estimating the between-study variance and using it to calculate the overall effect size. It provides a more conservative estimate of the average effect size. To assess the degree of heterogeneity between studies, the I^2^ statistic and the relative *p* value were taken into consideration, considering as significant the heterogeneity between studies in case of *p* < 0.10. The presence of possible publication bias was assessed by funnel plot [12].

The selected studies were divided into two groups, namely 177Lu- and 90Y-PRRT. The analysis of the pooled proportions was carried out on the DCR. The pooled descriptors of time-to-events (progression-free and overall survivals) in the various studies was determined by the algorithm suggested by Xiang Wan et al. [13] [sample-size weighted means ± standard deviation (SD)]. Descriptive statistics (means) with 95% confidence intervals (95% CI) were performed to pool and describe ages from different studies. Statistical analyzes were carried out using the R studio software version 4.1.1. (R studio Inc. Company, 250 Northern Avenue # 420 Boston, MA 02210, USA).

## 3. Results

Twelve studies met the criteria for this meta-analysis (from an initial identification of 5919 records) [14,15,16,17,18,19,20,21,22,23,24,25]: 10 with 177Lu-, 2 90Y- PRRT studies. The selection flow-chart and study characteristics are reported in Figure 1 and in Table 1 and Table 2, respectively.

In detail, we report pooled data on 149 177Lu- and 64 90Y-treated patients (total: 213 patients). Most studies were retrospective. The largest one included 46 patients (34 treated with 177Lu-, 12 with 90Y- PRRTs). Median ages ranged from 32.5 to 60.4 years. The female gender was slightly predominant (83 women, 74 men, gender not specified in 3 studies). When reported, mutations of *SDHB* (Succinate Dehydrogenase Complex Iron Sulfur Subunit B) were the most frequent genetic alterations. According to the predominant retrospective and real practice nature of the selected studies, sites of metastases and previous treatments were heterogeneous, with bone the metastatic site most often involved, and surgery the most frequent previous therapy (Table 1).

**Figure 2:**
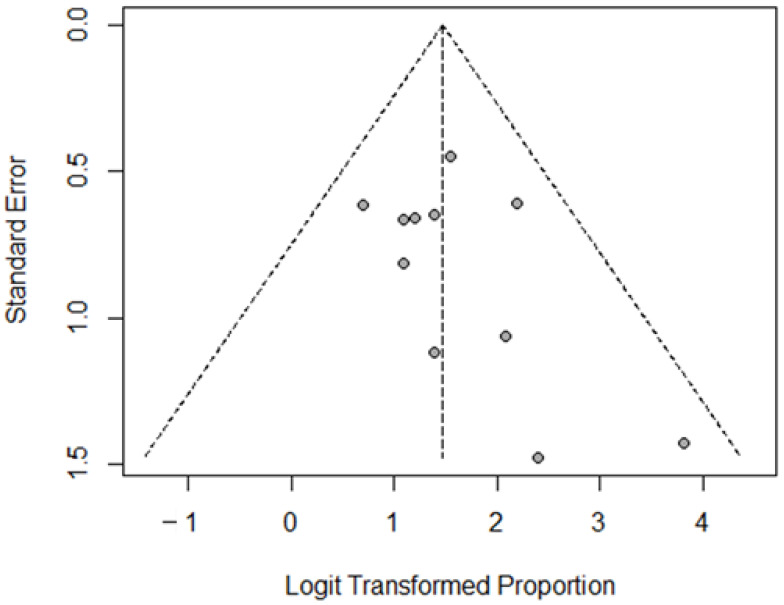
Funnel plot for DCR (disease-control rate) in the selected articles.

Paramount descriptive information on PRRT doses per cycle, number of cycles, response criteria, best responses to treatments, and frequency of G3/G4 hematologic toxicities is reported in Table 2. The lowest applied dose was 3.4 GBq per cycle; the number of administered cycles was heterogeneous (at least 3 cycles in 7 studies). The most used response criteria were the RECIST 1.1. Only one study evaluated the response with SWOG criteria, which are more conservative with regard to the definition of PR (50% or greater reduction in the sum of the product of the perpendicular diameters of target lesions). The majority of the patients experienced PR or SD. Hematologic toxicity was manageable. Funnel plot of selected studies did not show publication bias (Figure 2). The MINORS and Newcastle–Ottawa Scale scoring for the selected articles are reported in Table 3.

**Figure 3:**
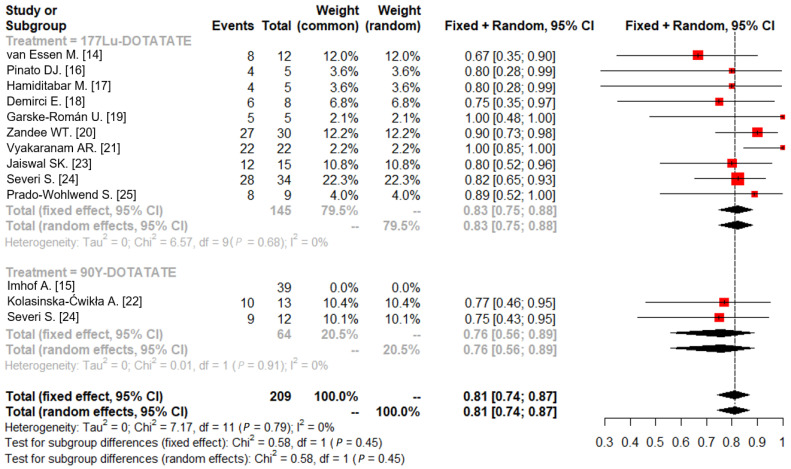
Forest plot for DCR according to 177Lu- and 90Y- PRRT treatments.

A forest plot of the treatments’ effect on DCR is shown in Figure 3. There was no statistically significant heterogeneity among the 177Lu- and 90Y- PRRTs studies (I^2^ value = 0%, *p* = 0.68 and I^2^ value = 0%, *p* = 0.91, respectively). The pooled DCRs were 0.83 (95% CI: 0.75–0.88) and 0.76 (95% CI: 0.56–0.89) for 177Lu- and 90Y-PRRT treatments, respectively. The pooled DCR for PRRT agents was 0.81 (95% CI: 0.74–0.87). Although the present study is the largest description of DCR in PRRT therapy of PCCs and PGLs, given the small sample size, no formal attempts were made to compare efficacy between 177Lu- and 90Y-PRRT treatments. 

Funnel and forest plots for response rate (complete plus partial responses) in the selected studies are reported in Figures S1 and S2, respectively. Table S1 synthetized patients’ age and time-to-event outcomes in the analyzed articles.

## 4. Discussion

PCCs and PGLs are rare neoplasms. For this reason, it is useful and legitimate to pool the effects from different studies in order to provide a more solid estimate of treatment efficacy. In the present meta-analysis, we show the efficacy of PRRT treatment based on 177Lu- DOTATATE and 90Y- DOTATOC in 213 patients affected by PCCs and PGLs. The primary endpoint was the correct quantification of DCR. We report a pooled DCR of 83% and 76% for 177Lu- DOTATATE and 90Y- DOTATOC treatments, respectively, and a good toxicity profile. Pooled estimates on response rates, age of enrolled patients and time-to-event are reported in a Supplementary File.

To our knowledge, this is the largest and first study reporting on the pooled effect of these specific treatments without radiosensitiziser administration. Radiosensitizers are agents that enhance the effectiveness of radiation therapy in the treatment of cancers. In the context of PRRT for pheochromocytomas and paragangliomas, radiosensitizers may be used to increase the uptake of radionuclides by tumor cells, to synergize with the anti-neoplastic effects, and to enhance the therapeutic efficacy of PRRT. However, the use of radiosensitizers in PRRT for PCCs and PGLs is still an area of ongoing research, and its role in clinical practice is unclear. A previous meta-analysis reported the pooled efficacy of PRRT in 201 patients with advanced PPCs and PGLs [26]. The authors included in their analysis clinical series treated with concomitant radiosensitizers (capecitabine/temozolomide/fluorouracil) and PRRT (concomitant 177Lu-DOTATATE and 90Y-DOTATOC). The use of different radiosensitizers can also produce spurious and scarcely comparable results.

The PRRT treatment achieved a DCR of 84% (95% CI: 77–89%), with similar responses among 90 Y- and 177 Lu-based agents. Non-hematologic toxicity was negligible. For this reason, we focused on hematologic toxic events. Another meta-analysis on PRRT (131I-MIBG) has been previously published [27]. It showed a high DCR (70%) but a high risk of publication bias, with inadequate clinical sample description and inadequate reporting of intervention and response criteria in 12 out 17 analyzed studies. Furthermore, a high rate of hematologic toxicity was evidenced (grade 3/4 neutropenia and trombocitopenia in 87% and 83% of patients, respectively).

Notably, there are no widely accepted guidelines for PCC and PGL therapy. In this context, a multidisciplinary approach is desired and pursued in most centers [8]. Surgery is applied in localized disease, while chemotherapy, radiotherapy, and PRRT are administered as palliative treatments in metastatic disease, where the goals are quality of life and survival improvement [28]. The role of chemotherapy is still controversial and debated. A meta-analysis on the role of cyclophosphamide, vincristine, and dacarbazine (CVD) as first-line treatments for metastatic disease in PCCs and PGLs evidenced a high pooled DCR (57%) assessed with RECIST 1.1, but a poor toxicity profile with a clinically significant discontinuation rate due to grade 3/4 episodes of hematological and non-hematological toxicities (neuropathy and gastro-intestinal symptoms) [29] highlighting the importance of patient selection. Unfortunately, there are neither large phase II nor randomized phase III trials validating the role of alternative chemotherapy schedules in this clinical setting.

To understand the therapeutic panorama of PCCs and PGLs, it is important to discuss that genetic assessments are gaining a crucial role in their diagnosis and management. These tumors are caused by mutations in specific genes, which can be inherited or occur sporadically. The characterization of the genetic background of these tumors will become increasingly important for diagnosis, prognosis, and treatment. The most common mutations associated with PCCs and PGLs are in the genes *RET*, *VHL*, *NF1*, *SDHA*, *SDHB*, *SDHC*, and *SDHD* [30]. These mutations result in alterations in the regulation of cell growth and division, leading to the development of the tumors. On a genetic point of view, PCCs and PGLs can be classified into two types: hereditary and sporadic. Hereditary forms are caused by mutations that are inherited from a parent and occur in multiple family members. The most common hereditary form is associated with mutations in the *RET* gene. Sporadic forms are caused by mutations that arise spontaneously and are not inherited from a parent. The majority of PCCs and PGLs are sporadic; in this case, *SDHB* is the most frequently mutated gene [31]. In the future, this characteristic could be exploited to target and cure these neoplasms. However, very few data are available on the role of targeted therapies. A phase II study analyzed the effect of sunitinib in patients with progressive PCCs or PGLs (SNIPP trial), reporting a promising DCR of 83% in 25 enrolled patients. Grade 3/4 toxicities were represented by fatigue (16%), thrombocytopenia (16%), cardiac events (8%), and hypertension (4%) [32]. Clinical studies on cabozantinib, axitinib, lenvatinib, and everolimus are ongoing, and available results are too few to provide definitive indications on the role of TKIs (tyrosin-kinases inhibitors) in PCCs and PGLs [33,34,35].

The PRRT treatment, with its manageable and low toxicity, represents a “less intensive” treatment. This finding is gaining great attention and re-evaluation in oncology, as these treatments refer to less aggressive therapeutic approaches in patients with high disease burdens not eligible for curative treatments (due to factors such as poor performance status, advanced age, or the presence of comorbid conditions). In this context, PRRT based on 177Lu-DOTATATE and 90-YDOTATOC without radiosensitizer is a highly valuable option.

The goal of this meta-analysis was the reporting of a solid and updated estimate of DCR achieved with 177Lu- and 90Y-PRRT in PCCs and PGLs. In this regard, the main limitations were the heterogeneity in response criteria (even if 10/12 studies use the RECIST) and the small sample size related to the rarity of the selected neoplasms. Another limitation of our study was the lack of registration in PROSPERO (an international database dedicated to the prospective registration of systematic reviews). There are two main consequences: a possible future work “duplication” and a potential bias related to the absence of comparison between what was methodologically planned in the protocol (at inception) and what was actually made and reported. These risks exist at any phase, including the review and post-publication periods. Even if, at present, there are no equivalent systematic reviews in the literature, registration in PROSPERO would have helped to avoid any effects of scientific redundancy of the researchers’ work.

## 5. Conclusions

We showed that PRRT based on 177Lu-DOTATATE and 90-YDOTATOC without radiosensitizers are efficacious therapeutic options (DCR of 83% and 76%, respectively) and can be considered in the multidisciplinary treatment of PCCs and PGLs as alternatives to I-131 MIBG and chemotherapy.

## Figures and Tables

**Table 1:** Baseline characteristics of included studies.

Author	Year	Type of Study	No. of Patients	Age, Years Median (Range)	Sex	Sites of Metastases (No. of Patients)	Genetic Characteristics (No. of Patients)	Previous Treatments (No. of Patients)
**van Essen M.** [14]	2006	retrospective	12 (1 PCC, 11 PGL)	39.7 (22–55)	6 male, 6 female	liver (4), bone (7)	NA	surgery (9), CHT (4), RT (7), naive (2)
**Imhof A.** [15]	2011	prospective	39 (11 PCC, 28 PGL)	39	NS	NS	NA	NS
**Pinato DJ.** [16]	2016	retrospective	5 (5 PGL)	34 (16–47)	4 male, 1 female	nodes (1), bone (5), lung (1)	SDHB (5)	surgery (4), CHT (1), RT (1), 131I-MIBG (1)
**Hamiditabar M.** [17]	2017	prospective	5 (1PCC, 4 PGL)	NS	NS	NS	NA	NS
**Demirci E.** [18]	2018	retrospective	12 (NS)	NS	NS	NS	NA	NS
**Garske-Román U.** [19]	2018	prospective	5 (2 PCC, 3 PGL)	60.4 (25–71)	2 male, 3 female	nodes (2), liver (2), bone (5)	NA	surgery (5)
**Zandee WT.** [20]	2019	retrospective	30 (3 PCC, 27 PGL)	47 (29–74)	10 male, 20 female	nodes (10), liver (7), bone (13), lung (6)	SDHB (5), SDHD (11), familiar (2), sporadic (5), unknown (7)	surgery (19), CHT (5), RT (6), 131I-MIBG (3), SSA (2)
**Vyakaranam AR.** [21]	2019	retrospective	22 (9 PCC + 13 PGL)	60 (24–80)	13 male, 9 female	nodes (7), liver (9), bone (17), lung (4)	sporadic (4), SDHB (4), SDHD (2), SDHA (1), NF1 (2), unknown (9)	surgery (16), CHT (1), RT (14), 131I-MIBG (6), naive (1)
**Kolasinska-Ćwikła A.** [22]	2019	prospective	13 (4 PCC + 9 PGL)	41.8 (27–62)	8 male, 5 female	liver (6), bone (9)	SDHB (5), SDHD (8)	surgery (13), SSA (8)
**Jaiswal SK.** [23]	2020	retrospective	15 (5 PCC + 10 PGL)	32.5	7 male, 8 female	nodes (3), liver (4), bone (6), lung (3)	VHL (2), SDHB (1), SDHD (1), negative (1), unknown (10)	surgery (10), RT (3)
**Severi S.** [24]	2021	retrospective	46 (NS)	52	20 male, 26 female	liver (8), bone (19)	wildtype (10), SDHD or SDHB (20)	NS
**Prado-Wohlwend S.** [25]	2022	retrospective	9 (3 PCC + 6 PGL)	45.8 (20–72)	4 male, 5 female	nodes (6), liver (3), bone (8), lung (3)	SDHD (1)—SDHB (3)—NF1 (1)—sporadic (4)	CHT (3), RT (3), 131I-MIBG (2), SSA (8), naive (1)

CHT: chemotherapy; NF1: neurofibromatosis type 1; NS: not specified; PCC: pheochromocytoma; PGL: paraganglioma; RT: radiotherapy; SDHA: succinate dehydrogenase subunit A; SDHB: succinate dehydrogenase subunit B; SDHD: succinate dehydrogenase subunit D; SSA: somatostatin analogues; VHL: von Hippel-Lindau.

**Table 2:** Efficacy and toxicity profile of PRRT in the included studies.

Author (First Name)	Year	PRRT Agent	Treatment Characteristics	No. of Cycles	Response Criteria	Best Response to PRRT (Radiologically Assessed Patients)	Haematological Toxicity Grades 3/4 (No. of Patients)
**van Essen M.** [14]	2006	177Lu	7.4 GBq per cycle	NS	SWOG	PR (2), SD (6)	thrombocytopenia and anemia (2)
**Imhof A.** [15]	2011	90Y	3.7 GBq/mq per cycle	2 (1–10)	RECIST 1.0	PR (7) *	NS
**Pinato DJ.** [16]	2016	177Lu	6.6–7.6 GBq per cycle	3	NS	PR (1), SD (3)	none
**Hamiditabar M.** [17]	2017	177Lu	7.4 GBq per cycle	NS	RECIST 1.1	SD (4)	NS
**Demirci E.** [18]	2018	177Lu	3.7–8.1 GBq per cycle	at least 3 cycles	RECIST 1.1	PR (4), SD (2)	NS
**Garske-Román U.** [19]	2018	177Lu	7.4 GBq per cycle	NS	RECIST 1.1	SD (5)	NS
**Zandee WT.** [20]	2019	177Lu	7.4 GBq per cycle	73% of patients received 4 cycles	RECIST 1.1	PR (7), SD (20)	anemia (2), thrombocytopenia (5), leukopenia (3)
**Vyakaranam AR.** [21]	2019	177Lu	7.4 GBq per cycle	4.9	RECIST 1.1	PR (2), SD (20)	no
**Kolasinska-Ćwikła A.** [22]	2019	90Y	3.4 GBq per cycle	NS (61% of patients received 2 cycles)	RECIST 1.0	PR (1), SD (9)	anemia (2)
**Jaiswal SK.** [23]	2020	177Lu	NS	4.13	RECIST 1.1	PR (1), SD (11)	none
**Severi S.** [24]	2021	177Lu (34) and 90Y- (12)	177Lu: 3.7–5.5 GBq per cycle 90Y: 1.1–1.85 GBq per cycle	5	RECIST 1.1	177Lu: PR (3), SD (25) 90Y: PR (1), SD (8)	none
**Prado-Wohlwend S.** [25]	2022	177Lu	8.01 (7.4–8.4) GBq per cycle	3.11	RECIST 1.1	PR (2), SD (6)	NS

GBq: Gigabecquerel; NS: not specified; PR: partial response; SD: stable disease. * In this study, the DCR is not evaluable.

**Table 3:** MINORS and Newcastle-Ottawa Scale scores of selected articles.

Author	Year	Accrual Time	MINORS Score	Newcastle Ottawa Scale Score			
				Selection	Comparability	Outcome	Total
**van Essen M.** [14]	2006	NS	7	3	0	3	6
**Imhof A.** [15]	2011	1997–2010	10	3	1	3	7
**Pinato DJ.** [16]	2016	2008–2014	9	3	0	3	6
**Hamiditabar M.** [17]	2017	2010–2016	8	3	0	3	6
**Demirci E.** [18]	2018	2010–2015	8	3	0	3	6
**Garske-Román U.** [19]	2018	2010–2014	8	3	1	3	7
**Zandee WT.** [20]	2019	2000–NS	10	3	1	3	7
**Vyakaranam AR.** [21]	2019	2005–2018	10	3	1	3	7
**Kolasinska-Ćwikła A.** [22]	2019	2006–2018	11	3	2	3	8
**Jaiswal SK.** [23]	2020	2010–2019	8	3	1	3	7
**Severi S.** [24]	2021	2008–2018	10	3	2	3	8
**Prado-Wohlwend S.** [25]	2022	2014–2021	9	3	1	3	7

NS: Not Specified.

## Data Availability

R studio software analysis for making Forest Plots is reported in https://zenodo.org/record/7621656#.Y-Os5i-ZO3A (accessed on 10 November 2022).

## Supplementary Materials

The following supporting information can be downloaded at: https://www.mdpi.com/article/10.3390/jcm12041494/s1. Figure S1: Funnel plot for response rate (complete plus partial responses) in the selected studies; Figure S2: Forest plot for response rate (complete plus partial responses) in the selected articles; Table S1: Pooled age and time-to-event in the analyzed articles.
