# Supplementary material for: Response to Peptide Receptor Radionuclide Therapy in Pheocromocytomas and Paragangliomas: A Systematic Review and Meta-Analysis
Source: J Clin Med. 2023 Feb 13;12(4):1494. doi: 10.3390/jcm12041494 (PMC9964778; doi:10.3390/jcm12041494)
Supplement: Supplementary file 1 [file jcm-12-01494-s001.zip › jcm-2059683-supplementary.pdf]

Journal of
Clinical Medicine

Systematic Review

Response to peptide receptor radionuclide therapy in pheocromocytomas and paragangliomas: a systematic review and meta-analysis.

Supplementary

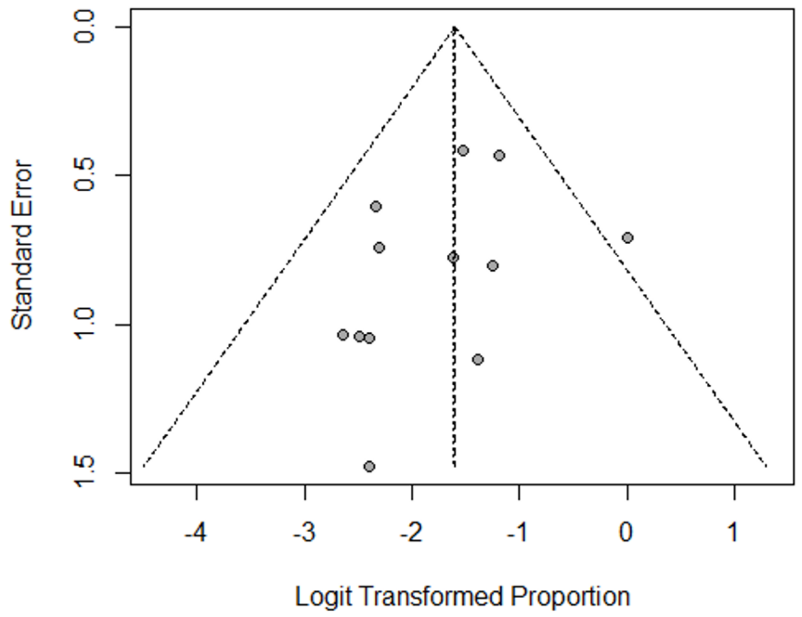

Figure S1. Funnel plot for response rate (complete plus partial responses) in the selected studies.

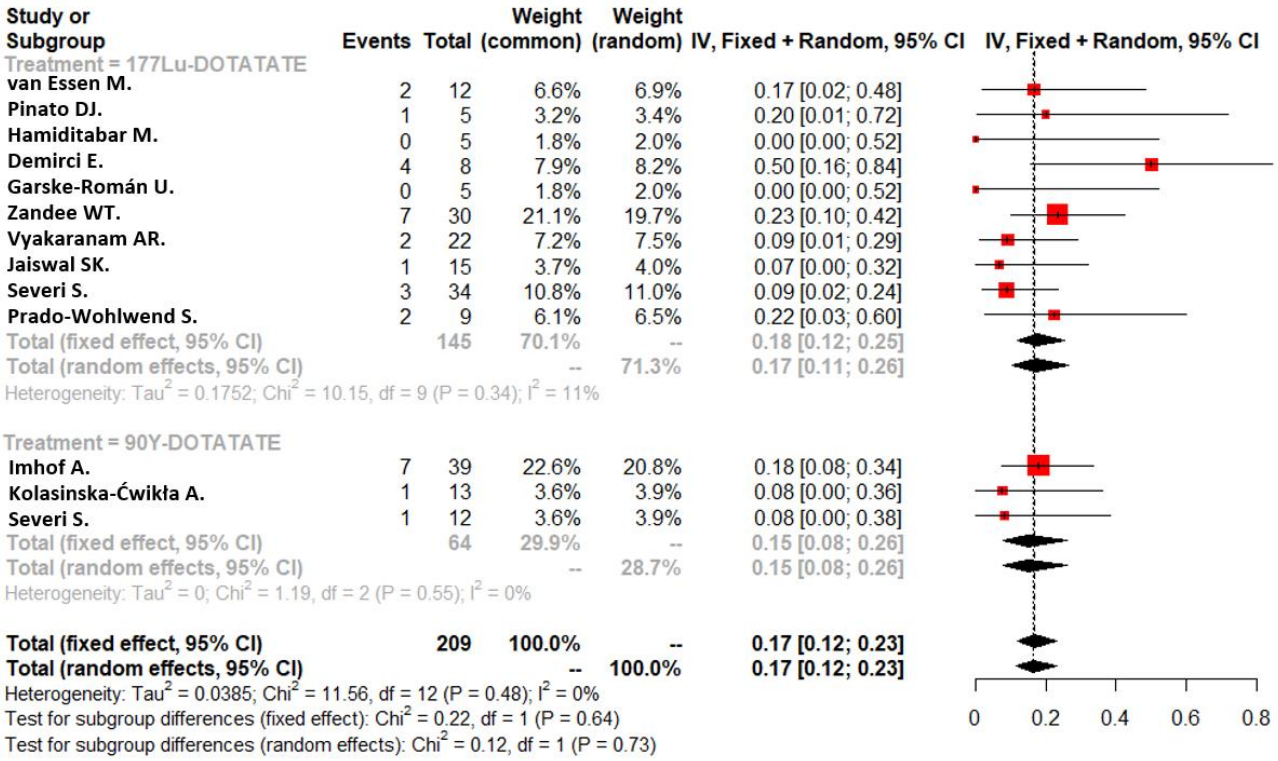

Figure S2. Forest plot for response rate (complete plus partial responses) in the selected articles.

Table S1. Pooled age and time-to-event in selected studies.

Characteristic	Articles assessed	Treatment	
		**177LU-DOTATATE**	**90Y-DOTATOC**
**Patients' age (years), mean (95% CI)**	Garske-Román U. *et al.*<br>Jaiswal SK. *et al.*<br>Pinato DJ. *et al.*<br>Prado-Wohlwend S. *et al.*<br>Vyakaranam AR. *et al.*<br>Zandee WT. *et al.*	46.42 (38.62-54.22)	Incalculable*
**Progression-free survival (months)**	Demirci E. *et al.*<br>Garske-Román U. *et al.*<br>Jaiswal SK. *et al.*<br>Pinato DJ. *et al.*<br>Prado-Wohlwend S. *et al.*<br>Vyakaranam AR. *et al.*<br>Zandee WT. *et al.*	24.5 ± 6.5	Incalculable*
**Overall survival (months)**	Demirci E. *et al.*<br>Garske-Román U. *et al.*<br>Pinato DJ. *et al.*<br>Vyakaranam AR. *et al.*	48.9 ± 4.7	Incalculable*

*There were no sufficient data to draw pooled age and/or time-to-event in 90Y-DOTATATE studies.
CI: Confidence Intervals.
